# Transforming Feather Meal Into a High‐Performance Feed for Broilers

**DOI:** 10.1002/vms3.70199

**Published:** 2025-01-15

**Authors:** Mandana Salehizadeh, Maryam Tajabadi Ebrahimi, Seyed Naser Mousavi, Abbas Akhavan Sepahi, Reza Orooji

**Affiliations:** ^1^ Department of Biology Science and Research Branch Islamic Azad University Tehran Iran; ^2^ Department of Biology, Faculty of Sciences, Central Tehran Branch Islamic Azad University Tehran Iran; ^3^ Department of Animal Science Varamin‐Pishva Branch Islamic Azad University Varamin Iran; ^4^ Department of Microbiology North Tehran Branch, Islamic Azad University Tehran Iran; ^5^ Department of Industrial Management Faculty of Humanities University of Tehran Kish International Campus Tehran Iran

**Keywords:** broiler chicken, feather meals, nutrient digestibility, performance, *Streptomyces* bacteria

## Abstract

**Background:**

The poultry industry faces challenges with the high cost and environmental impact of Soybean meal. Feather meal, a byproduct with low digestibility due to its keratin content, is a potential alternative. Recent biotechnological advances, including enzymatic and bacterial hydrolysis, have enhanced its digestibility and nutritional value.

**Objective:**

This study aimed to evaluate and compare the efficacy of three different feather meal processing methods as partial replacements for soybean meal in broiler diets. The methods assessed included hydrolyzed feather meal treated with *Streptomyces* bacteria, commercial enzyme‐treated feather meal, and boiled feather meal. Their effects on performance and nutrient digestibility were evaluated in Ross 308 male broiler chickens.

**Materials:**

The study involved 525 Ross 308 male broiler chickens were allocated to seven dietary treatments, which included a control diet and varying combinations of feather meal and soybean meal. *Streptomyces* sp. MSM5 was shown to effectively produce keratinase, enhancing the amino acid content of the feather meal.

**Results:**

Birds fed diets containing 33% and 67% boiled feather meal exhibited significantly reduced body weight gain (BWG) and increased feed conversion ratio (FCR) compared to the control group (*p* < 0.05). In contrast, birds fed diets with 33% hydrolyzed feather meal, treated with either enzymes or bacteria, showed similar BWG and FCR to those on the control diet. However, diets with 67% hydrolyzed feather meal led to significant reductions in performance (*p* < 0.05).

Additionally, diets containing 33% and 67% boiled feather meal, as well as 67% hydrolyzed feather meal, resulted in significantly lower carcass percentage, breast muscle percentage, thigh muscle percentage, overall dry matter digestibility, and protein digestibility (*p* < 0.05).

**Conclusion:**

The results indicate that substituting as much as 33% of soybean meal with processed feather meal, particularly when treated with enzymes or bacteria, does not negatively impact broiler performance. Furthermore, the results underscore the potential of biotechnological treatments, such as bacterial keratin hydrolysis, to enhance the nutritional value of feather meal, transforming it into a high‐quality, sustainable protein source for the poultry industry.

## Graphical abstract

The study evaluated the effectiveness of three feather meal processing methods as soybean meal replacements in broiler diets. Hydrolyzed feather meal treated with enzymes or bacteria showed similar performance to the control, while boiled feather meal significantly reduced bird growth and digestibility. Biotechnology‐enhanced feather meal offers a sustainable, high‐quality protein alternative for poultry.

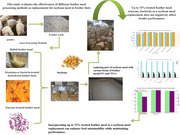

## 1 Introduction

Feathers, a byproduct of poultry processing, consist primarily of keratin protein (∼90%). If not managed properly, the accumulation of feathers poses environmental challenges, contributing to pollution and wasting a valuable protein resource. Utilizing feathers as animal feed offers a dual benefit: it is both cost‐effective and eco‐friendly, reducing waste disposal needs and production expenses (Grazziotin et al. 2006; Adejumo and Adetunji 2018). Given that feed costs account for up to 70% of poultry production expenses, finding economical protein alternatives is essential to alleviating the financial burden on producers. Feather meal, a protein‐rich feed ingredient, has been proposed as a partial replacement for conventional protein sources such as soybean meal, which are costly and contribute to rising feed prices (Alshelmani et al. 2021). However, feather protein is poorly digested by monogastric animals due to its keratinized structure (Kim, Lorenz, and Patterson 2002). To enhance its digestibility, various processing methods, including hydrolysis and enzyme treatment, have been explored (Ochetim 1993). Among these, keratinase enzymes, produced by fungi, actinomycetes, and bacteria such as *Bacillus subtilis*, have demonstrated promising potential in degrading keratin‐rich substrates (Călin et al. 2017; Alshelmani et al. 2024). Feather hydrolysates derived from microbial keratinase activity, such as those from *Bacillus licheniformis* PWD‐1 and *Vibrio* sp. strain kr2, have been successfully used as feed additives to enhance nutrient availability and digestibility (Williams et al. 1991; Grazziotin et al. 2006). Boiling feathers under steam pressure is another widely used processing technique, allowing feather meal to partially replace soybean meal in broiler diets without negative effects on performance (Ochetim 1993). Despite these advances, limitations remain in terms of nutrient retention, processing efficiency, and the scalability of biotechnological approaches.

This study addresses key limitations in feather meal utilization by exploring innovative processing methods, including hydrolysis with keratinase derived from *Streptomyces* bacteria, enzyme‐assisted hydrolysis, and steam‐pressure boiling. These advanced techniques aim to enhance the nutritional quality, digestibility, and amino acid profile of feather meal, making it a more viable and sustainable protein source for broiler chickens. By examining how these processing methods can improve the efficiency of feather meal as a feed ingredient, the study fills an important gap in current knowledge. Ultimately, it offers valuable insights for optimizing feather meal processing, contributing to cost‐effective and environmentally friendly poultry production. From an industrial standpoint, this research provides practical guidance on reducing reliance on expensive protein sources such as soybean meal, presenting a promising alternative that supports both economic sustainability and waste reduction in the poultry industry.

## 2 Materials and Methods

### 2.1 Isolation and Purification of Keratinase Producing Bacteria

Poultry‐deteriorated soil samples were collected from Saveh's poultry farms near Markaze province in September 2022. A 10‐g soil sample was mixed with 90 mL of phosphate buffer (50 mM, pH 7.0) and shaken at 150 rpm for 30 min to dislodge microbial cells. The resulting suspension was serially diluted to a 10^−10^ dilution factor. From each dilution, 100 µL was spread onto modified feather meal agar plates, where feather meal served as the sole carbon and nitrogen source. Feather meal was prepared by washing poultry feathers thoroughly with water to remove debris, followed by drying at 60°C. The dried feathers were milled into a fine powder and sterilized at 121°C for 15 min before incorporation into the medium. The modified feather meal agar medium was composed of 0.5 g/L NaCl, 0.7 g/L KH_2_PO_4_, 1.4 g/L K_2_HPO_4_, 0.1 g/L MgSO_4_, and 10 g/L feather meal, adjusted to pH 10.0 to favour the growth of alkalophilic microorganisms. The plates were incubated at 37°C for 48–72 h, until visible colonies formed. Bacterial colonies producing keratinase were identified based on clear zones around colonies, indicative of keratin degradation. Identification of the isolates was carried out according to *Bergey's Manual of Systematic Bacteriology*, using microscopic examination and biochemical tests (Haq, Akram, and Jabbar 2020).

### 2.2 Preparation of Keratin Substrate for Enzyme Activity Measurement

To evaluate keratinase activity, dimethyl sulfoxide (DMSO)‐solubilized keratin and a prepared seed culture were utilized. DMSO was employed for its ability to effectively dissolve keratin, stabilize the enzyme and substrate, improve their interaction, and ensure precise experimental control. This is particularly important for working with insoluble substrates such as keratin.

### 2.3 Preparation of Keratin–DMSO Solution

Ten grams of cleaned feathers were used to prepare a keratin–DMSO solution. The feathers were thoroughly washed with detergent to remove debris, oils, and contaminants, then repeatedly rinsed with distilled water and air‐dried. The cleaned feathers were mixed with 500 mL of DMSO and heated at 100°C for 120 min to solubilize the keratin. Then, 1000 mL of cold acetone was added to the mixture, which was then incubated at −24°C for 5 h to precipitate the keratin. The precipitate was rinsed, dried, and dissolved in 20 mL of 0.05 N NaOH. Finally, the pH of the solution was adjusted to 8 using 0.1 mol/L Tris‐HCl, resulting in the final keratin‐DMSO solution (Mehta, Jholapara, and Sawant 2014).

### 2.4 Pure Enzyme Preparation (Seed Culture)

Selected bacterial colonies cultured on nutrient agar were transferred to Minimal Salt Medium with 1% feather as the sole carbon and nitrogen source. After 48–72 h of incubation, the supernatant was collected as a crude enzyme solution (Mehta, Jholapara, and Sawant 2014).

### 2.5 Keratinase Measurements

Keratinase activity was measured by incubating 1 mL of crude enzyme with 1 mL of keratin solution at 50°C for 10 min. The reaction was stopped with 20% trichloroacetic acid, and the absorbance of the supernatant at 280 nm was measured to determine enzyme activity.

The enzyme activity (U) was calculated using the formula:

(1) U=4×n×A280

where 0.01 represents the absorbance number indicated by the control, *n* is the dilution number, 4 is the final volume in millilitres, and 10 is the incubation duration in min. One unit of enzyme activity is defined as the amount that increases the optical absorbance by 0.01 under the same conditions. Keratinase activity is reported in units/mL/min. The negative control used was the culture medium without the substrate (feather) (Mehta, Jholapara, and Sawant 2014).

### 2.6 Determination of Percentage of Keratin Feather Degradation

To isolate keratinolytic bacteria, cultures were grown overnight in 10 mL nutrient medium from agar slants at 37°C. These were then used to inoculate 50 mL nutrient medium in Erlenmeyer flasks with 1% whole chicken feathers. Incubation lasted up to 96 h at 30–50°C with agitation. Feather degradation was monitored visually at 6‐h intervals. After incubation, remaining feathers were filtered, washed, dried, and weighed to calculate degradation percentage.

(2) Percentageofdegradation=TWF−RWF/TWF×100,

where TWF is total weight of feathers and RWF is residual weight of feathers.

Keratinolytic bacterial isolates showing maximum feathers degradation were selected for further experimental studies (Kurane and Attar 2017).

### 2.7 Preparation of Keratinase Hydrolysis Bacteria

After cultivating bacteria (∼10^8^ CFU/mL), feathers were mixed with growth medium (250 g feathers/L) in a fermentation vessel. The medium was nitrogen‐flushed and incubated aerobically for 5 days at 50°C under constant agitation (120 rpm/min). The mixture was then autoclaved at 125°C for 15 min, dried at 60°C for 48 h, and milled through a 1‐mm mesh. The resulting brown powder, containing partially hydrolyzed feathers, peptides, amino acids, and inactivated bacteria, had a keratinase activity of 23.43 U/mL and was used as a dietary protein source for chickens (Adejumo and Adetunji 2018).

### 2.8 Production of Hydrolyzed Feather Meal

At the Sepedbal Saveh slaughterhouse, 30 tons of raw chickens are processed daily, consisting of 8% feathers, 5% intestines, and 1% blood. In industrial‐scale production, 2.4 tons of feathers and 800 L of water are separated and added to a 10‐ton capacity cooker. In the feather meal production process, concentrated crude bacterial keratinase (1.5 kg crude enzyme for every 1000 kg of wet material) and commercial enzyme (1.5 kg Valkerase^1^ for every 1000 kg of wet material) are separately introduced into a cooker/drier. The mixture incubated at 60°C for 1 h, then autoclaved at 130°C at 1.8–1.9 bars for 20 min, with pressure gradually declining over 60 min. Post‐hydrolysis, feathers were dried to 10% moisture content and hammer‐milled to 3‐mm particles. The feather meal was stored in a silo and analyzed for protein, pepsin digestibility, and amino acid profiles (Tables 2 and 4).

### 2.9 Pepsin Digestibility

Pepsin‐digestible nitrogen was determined using the AOAC (2005) procedure. Prior to analysis, a 0.5‐g sample was prepared by finely grinding or homogenizing the material to ensure uniformity. The sample was then incubated in 150 mL of 0.2% pepsin solution (1:10,000 activity) in 0.075 N HCl at 45°C for 16 h. During this incubation, triplicate samples were agitated periodically to maintain the required temperature and ensure thorough digestion (SabahelKhier, Hussain, and Ishag 2010).

The pepsin digestibility was calculated using the formula:

(3) $$\mathrm{Pepsin}\;\mathrm{digestibility}\;\left(\%\right)=\mathrm{Pepsin}-\mathrm{digestible}\;\mathrm{nitrogen}\;\left(mg\right)$$

Total nitrogen (mg)

 where pepsin‐digestible nitrogen (mg) is the amount of nitrogen digested by pepsin after incubation and total nitrogen (mg) is the initial total nitrogen content in the sample before digestion.

### 2.10 Amino Acid Analysis

Amino acid analysis was performed by ion‐exchange chromatography (Manning et al. 1972). The amino acids were obtained by peptide hydrolysis with 6MHCl at 110°C for 24 h and purified with Amberlite IR‐120.

(4) $$\begin{array}{c} \mathrm{Amino}\;\mathrm{acid}\;\mathrm{content}\left(g/100\;g\mathrm{CP}\right)\mathrm{of}\;\mathrm{treatment}\\ \times \;\left(100-N\;\mathrm{solubility}\;\mathrm{of}\;\mathrm{treatment}\right)\\ \mathrm{amino}\;\mathrm{acid}\;\mathrm{content}\left(g/100g\;\mathrm{CP}\right)\mathrm{of}\;\mathrm{control}\\ \times \;(100-N\;\mathrm{solubility}\;\mathrm{of}\;\mathrm{control}) \end{array}$$

- Amino acid content (g/100 g CP) of treatment: This is the value you are calculating. It represents the amino acid content in the treated sample per 100 g of crude protein (CP).
- Amino acid content (g/100 g CP) of control: This value represents the baseline or control sample's amino acid content per 100 g of crude protein.

### 2.11 Birds, Management, and Treatments

A total of 525 one‐day‐old Ross 308 male broiler chickens were obtained from a local supplier and randomly allocated to seven dietary treatments, with three replicates of 25 birds each, and reared up to 42 days. Feed and water were provided ad libitum.

The initial room temperature was set at 32°C, which was gradually decreased by 2°C per week until reaching 22°C at 5 weeks of age. Once 22°C was achieved, the temperature was maintained with minimal fluctuations (±1°C). Temperature was continuously monitored using digital thermometers placed at multiple locations in the room to ensure uniform distribution of heat.

Ambient relative humidity was maintained at 55 ± 5% throughout the study period. Humidity was closely monitored using a digital hygrometer to ensure optimal growing conditions.

Light was provided according to a 23L:1D photoperiod (23 h of light and 1 h of darkness daily), and this photoperiod was maintained throughout the study period from 1 to 42 days. Light sources used were LED lights, emitting light with a colour temperature of with a colour temperature of 5000 K and maintaining intensity at 300 lux at the bird level.

The birds were housed in floor pens measuring 2 m^2^, with wood shavings used as litter material. The litter depth was maintained at 10 cm and was regularly turned and replenished with fresh material to absorb moisture and minimize ammonia buildup. This management practice is essential for preventing leg health issues, such as footpad lesions and lameness. The stocking density was set at 10 birds per m^2^, with each pen, which measured 2 m^2^, housing 25 birds. This resulted in a stocking density of 12.5 birds per m^2^. The stocking density was determined based on the space required for the birds, considering factors such as available floor area, access to feed and water, and the placement of pen equipment, including feeders and drinkers. Round feeders with a 30‐cm diameter were provided, and nipple drinkers, with one per 10 birds, were installed to ensure adequate access to both feed and water.

Birds were vaccinated for Newcastle disease virus on day 8 and 18, for infectious bronchitis on day 1 and 14, and for Gumboro disease on day 15 and 24.

A basal diet, formulated to meet the nutritional requirements outlined in the Ross Broiler Nutrition Specification (2018, Table 1), was used as the control diet. The experimental treatments were formulated by replacing part of the soybean meal with various forms of feather meal. Seven dietary treatments were used, each with three replicates of 25 birds per treatment (Adejumo and Adetunji 2018).

**TABLE 1:** Ingredients and composition of the basal diet.

Ingredients	Starter	Grower	Finisher
Corn	54	57	63
Soybean meal (42% CP)	39.22	37.01	31.18
Soybean oil	1.63	2.33	2.39
Dicalcium phosphate	1.7	1.31	1.01
Calcium CO_3_	1.07	0.78	0.73
NaHCO_3_	0.1	0.1	0.1
Salt (NaCl)	0.29	0.29	0.29
Vitamin—Mineral premix^2^	0.5	0.5	0.5
l‐Lysine HCL	1.05	0.27	0.43
DL‐Methionine (98%)	0.41	0.37	0.32
Calculated composition			
Metabolisable energy (kcal/kg)	2950	3025	3090
Crude protein (%)	22.5	21	19
Calcium (%)	0.9	0.7	0.6
Available phosphorous (%)	0.45	0.38	0.32
Sodium (%)	0.16	0.16	0.16
Lysine (%)	2.2	1.38	1.39
Methionine (%)	0.71	0.67	0.59
Methionine + Cystine (%)	1.05	1	0.9
Threonine (%)	0.82	0.79	0.71

The dietary treatments were as follows:

1. Control diet (C): No feather meal replacement.
2. 33% Boiled feather meal (33BFM): 33% boiled feather meal + 67% soybean.
3. 67% Boiled feather meal (67BFM): 67% boiled feather meal + 33% soybean.
4. 33% Hydrolyzed feather meal with commercial enzyme (33HFME): 33% hydrolyzed feather meal with commercial enzyme + 67% soybean.
5. 67% Hydrolyzed feather meal with commercial enzyme (67HFME): 67% hydrolyzed feather meal with commercial enzyme + 33% soybean.
6. 33% Hydrolyzed feather meal with *Streptomyces* bacteria (33HFM‐S): 33% hydrolyzed feather meal with *Streptomyces* bacteria + 67% soybean.
7. 67% Hydrolyzed feather meal with *Streptomyces* bacteria (67HFM‐S): 67% hydrolyzed feather meal with *Streptomyces* bacteria + 33% soybean.

We are pointing out that typically in poultry diets, soybean content is between 23% and 35% of the total feed, and we want to clarify that when the treatment contains 67% feather meal, the remaining portion (33%) of the diet is soybean. So, we want to be clear that the percentages of soybean and feather meal are being used to describe the relative proportion of those two ingredients within the treatment, and the rest of the diet is made up of other ingredients (e.g., grains, vitamins, minerals).

### 2.12 Growth Performance and Relative Organ Weights

Growth performance parameters, including body weight gain (BWG), feed intake (FI), and feed conversion ratio (FCR), were monitored weekly during the study phases: starter (1–11 days), grower (11–24 days), finisher (24–42 days), and overall (1–42 days). BWG was calculated as the difference in body weight between consecutive weeks, FI was recorded by measuring feed consumed weekly, and FCR was determined as the ratio of feed intake to BWG. Mortality was recorded daily and used to adjust FCR calculations. At the study's conclusion, relative organ weights were measured by euthanizing two birds per replicate, with organs such as the liver, gizzard, heart, and spleen excised and weighed. Relative organ weights were calculated as a percentage of live body weight. Carcass yield was determined by measuring the weight of the whole carcass after evisceration, and carcass portions (breast, thigh, and drumstick) were weighed as a percentage of live body weight.

### 2.13 Apparent Ileal Digestibility of Nutrients

Nutrient digestibility was assessed in the final 3 days of the experiment using 3 g/kg of titanium dioxide (TiO_2_) as an indigestible marker. Two birds from each replicate were humanely euthanized through cervical dislocation followed by severing the jugular vein.

Ileal contents (between Meckel's diverticulum and 2 cm above the ileocecal junction) were collected and stored at −20°C (Alshelmani 2015; Alshelmani et al. 2016, 2017). Samples were analyzed for dry matter (DM) by drying at 105°C for 24 h and subsequent ashing at 600°C. Crude protein (CP) was determined by the Kjeldahl method, ether extract (EE) by Soxhlet extraction (AOAC 2005), calcium (Ca) by complexometric titration, and phosphorus (P) by the vanadium‐molybdate method (AOAC 2005). TiO_2_ concentration in diets and digesta was analyzed, and its absorption was measured spectrophotometrically at 410 nm. Nutrient digestibility (ND) was calculated using the established equation (Cho et al. 2011; Faryadi et al. 2021).

ND (%) = 1 − [(TiO_2_ in diet/TiO_2_ in sample) × (Nutrient in sample/Nutrient in diet)] × 100. (5)

### 2.14 Statistical Analysis

The data were analyzed using the GLM procedures of SAS Support (2002) completely randomized design (CRD). Differences among treatments means were compared using Duncan's multiple range tests. Statistical significance was considered at *p* < 0.05.

## 3 Results

The crude protein content and pepsin digestibility of treatments containing hydrolyzed feather meal are shown in Table 2. The crude protein content and pepsin digestibility of hydrolyzed feather meal treated with enzyme and *Streptomyces* sp. MSM5 were significantly higher than those of untreated feather meal (*p* < 0.05). Enzymatic treatment resulted in the highest crude protein content (61.30%) and pepsin digestibility (30.92%), followed by bacterial treatment with a crude protein content of 59.76% and pepsin digestibility of 29.66%. In contrast, untreated feather meal showed the lowest values, with crude protein content at 57.50% and pepsin digestibility at 21.70%. The differences in both crude protein content and pepsin digestibility among treatments were statistically significant (*p* < 0.05). This indicates that enzymatic and bacterial treatments effectively improve the protein content and digestibility of feather meal.

**TABLE 2:** Crude protein content and pepsin digestibility of soybean protein, feather meal, and enzymatic feather and bacterial feather.

Iitem	Treatment				
	Feather meal	Enzymatic feather	Bacterial feather	SEM	*p* value
Crude protein (%)	57.50^b^	61.30^a^	59.76^a^	0.61	0.007
Pepsin digestibility (%)	21.70^b^	30.92^a^	29.66^a^	1.49	0.0002

*Note*: In each row, means with different superscripts are significantly different (*p* < 0.05).

Abbreviation: SEM, standard error of means.

**FIGURE 1:**
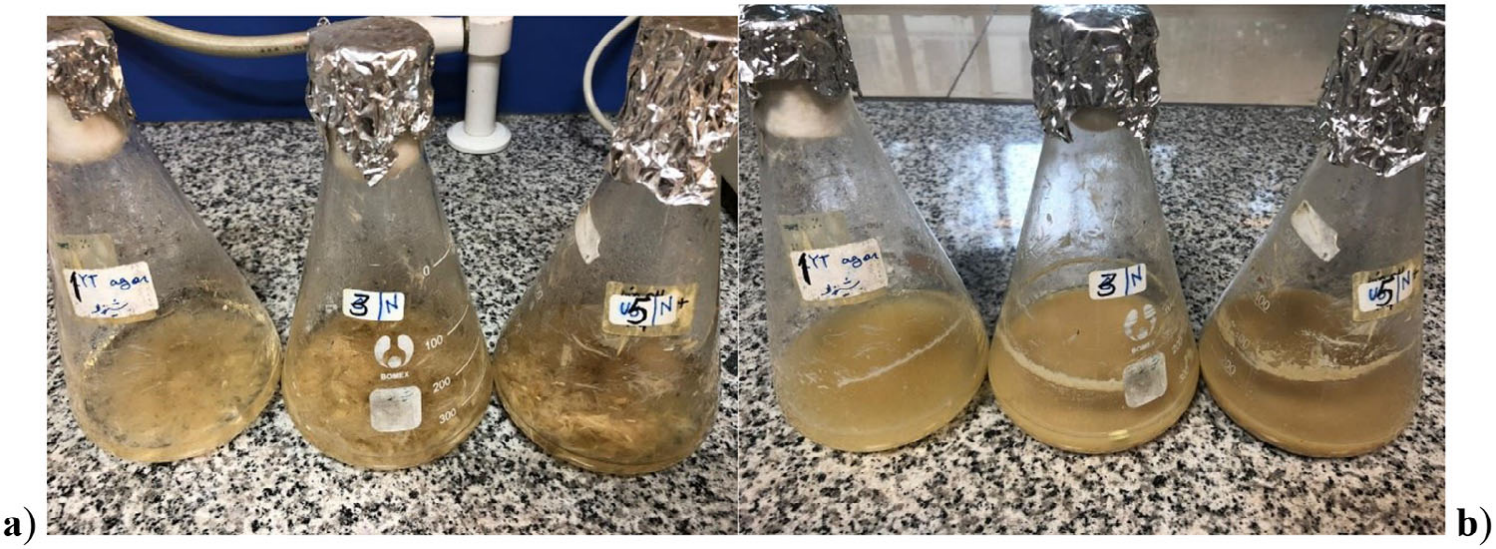
Biodegradation of chicken feather by *Streptomyces* sp. Strain MSM5: (A) before incubation and (B) after 96 h of incubation.

The keratinase production of *Streptomyces* bacteria is illustrated in Table 3. After culturing the bacteria, *Streptomyces* sp. MSM5 exhibited higher keratinolytic activity compared to the other strains, as shown in Figure 1. Isolate MSM5 demonstrated the highest keratinolytic activity, with the largest zone of clearance (2.5 cm), the greatest feather degradation (67.64%), and the highest enzyme activity (23.43 U/mL), making it the most effective isolate for feather degradation. These results suggest that MSM5 possesses the highest keratinolytic potential among the isolates, showing superior feather degradation and enzyme activity, which makes it a promising candidate for applications involving feather degradation.

**TABLE 3:** Evaluation of keratinolytic activities of the study bacterial isolates with the formation of zone of clearance on feather agar meal, degree of degradation, and enzyme activity.

Isolates	DZCon FAM medium (cm)	Feather degradation (%) DD%	Enzyme activity (U/mL)
MSM1	1.0de	52.33d	9.44ed
MSM2	1.20cd	52.66d	10.20cd
MSM3	0.50e	35.60g	6.33g
MSM4	1.70bc	56.04bc	11.62cb
MSM5	2.50a	67.64a	23.43a
MSM6	1.26bcd	52.84d	9.14def
MSM7	1.0de	51.83d	9.24def
MSM8	1.0de	51.41d	9.77d
MSM9	1.23bcd	52.13d	9.50de
MSM10	0.53e	36.11fg	7.23g
MSM11	1.80b	57.41b	12.30b
MSM12	0.90de	37.06efg	7.70gf
MSM13	0.96de	37.81ef	6.76g
MSM14	0.86de	38.11e	7.90gef
MSM15	1.70bc	55.05c	10.63bcd

*Note*: Diameter of zone of clearance on feather agar meal (FAM) medium (cm), DZC on FAM medium.

Abbreviation: DD (%), degree of degradation (%).

The amino acid compositions of the feather meals are presented in Table 4. The alanine, cysteine, glycine, serine, and valine levels of hydrolyzed feather meal with enzyme and *Streptomyces* sp. MSM5 were significantly higher than soybean and boiled feather meal. However, lysine, methionine, histidine, and tryptophan contents of soybean were significantly higher than the other treatments. Additionally, the effects of hydrolyzed feather meal with enzymes and *Streptomyces* sp. MSM5 were better than the boiled feather meal. The results showed that enzyme and *Streptomyces* sp. MSM5 treatments could increase the amino acid content in the feather meal.

**TABLE 4:** Amino acid composition of soybean, feather meal, enzymatic feather, and bacterial feather.

	Treatment					
Amino acid (g/100 g)	Soybean	Feather meal	Enzymatic feather	Bacterial feather	SEM	*p* value
Alanine	4.44b	4.91a	5.23a	5.20a	0.1	0.0004
Lysine	6.22a	4.40b	4.69b	4.68b	0.21	0.0001
Arginine	6.27	6.1	6.19	6.25	0.02	0.07
Cysteine	1.90c	3.55b	4.41a	4.51a	0.31	0.0001
Glycine	4.26c	6.27b	7.78a	7.92a	0.44	0.0001
Histidine	2.63a	0.75b	0.87b	0.84b	0.23	0.0001
Isoleucine	4.78	4.76	4.93	4.81	0.03	0.2
Leucine	7.01	7.05	7.12	7.12	0.01	0.08
Methionine	1.40a	0.43c	0.76b	0.66b	0.1	0.0001
Phenylalanine	5.01	4.78	4.8	4.95	0.03	0.04
Serine	5.74c	10.59b	11.62a	11.55a	0.73	0.0001
Threonine	4.22	4.1	4.08	4.19	0.03	0.38
Tryptophan	1.40a	0.74c	0.82b	0.82b	0.08	0.0001
Tyrosine	3.6	3.35	3.43	3.52	0.04	0.31
Valine	4.70c	6.96b	7.53a	7.61a	0.35	0.0001

*Note*: In each row, means with different superscripts are significantly different (*p* < 0.05).

Abbreviation: SEM, standard error of means.

The effects of different forms and levels of feather meal on performance are presented in Table 5. Supplementing the diet with various forms of feather meal did not significantly affect BWG, FI, or FCR during the starter (1–10 days) and grower (11–21 days) phases (*p* > 0.05). However, during the finisher phase (22–42 days) and the overall trial period (1–42 days), birds fed diets containing 33% or 67% boiled feather meal (33BFM, 67BFM) showed a significant decrease in BWG and a corresponding increase in FCR compared to the control group (C) (*p* < 0.05). In contrast, diets containing 33% hydrolyzed feather meal with commercial enzyme (33HFME) or with *Streptomyces* bacteria (33HFM‐S) did not differ significantly from the control in terms of BWG or FCR during the finisher phase or the overall trial period (1–42 days). However, diets with 67% inclusion of either hydrolyzed feather meal with enzyme (67HFME) or bacteria (67HFM‐S) resulted in a significant decrease in BWG and an increase in FCR. Additionally, the performance of birds fed 33% hydrolyzed feather meal with enzymes (33HFME) was significantly better than that of birds fed 33% hydrolyzed feather meal with *Streptomyces* bacteria (33HFM‐S) (*p* < 0.05). These results suggest that hydrolyzed feather meal with enzyme is more beneficial for bird performance compared to hydrolyzed feather meal with bacteria, especially at higher inclusion levels.

**TABLE 5:** The effect of different forms and levels of feather on performance in broilers.

	Dietary treatments								
		Feather meal		Enzymatic feather		Bacterial feather			
Item	Control	33	67	33	67	33	67	SEM	*p* value
**1–10 days**									
Body weight	223.66	220.73	221.93	221.71	229.11	222.33	221.93	0.94	0.28
Feed intake	225.85	231.41	232.06	234.74	233.09	233.65	235.72	1.29	0.55
FCR	1.00	1.04	1.04	1.05	1.01	1.04	1.06	0.007	0.29
**11–21 days**									
Body weight	599.17	573.84	593.77	595.93	617.03	592.26	592.43	4.94	0.51
Feed intake	893.52	914.18	928.79	897.85	907.35	916.04	913.83	4.79	0.56
FCR	1.49	1.59	1.56	1.50	1.47	1.54	1.54	0.01	0.23
**22–42 days**									
Body weight	1813.06a	1711.06b	1717.73b	1815.76a	1720.23b	1810.06a	1716.75b	11.89	0.0002
Feed intake	3282.78	3312.52	3296.61	3275.85	3266.41	3233.63	3289.71	7.70	0.14
FCR	1.81b	1.93a	1.91a	1.80b	1.89a	1.78b	1.91a	0.01	0.0006
Body weight	2635.89a	2505.64c	2533.43c	2633.40a	2566.38b	2624.65a	2531.12c	11.88	0.0001
Feed intake	4402.15	4458.11	4457.47	4408.45	4406.86	4383.33	4439.26	8.83	0.12
FCR	1.67c	1.77a	1.75a	1.67c	1.71b	1.67c	1.75a	0.01	0.0001

*Note*: In each row, means with different superscripts are significantly different (*p* < 0.05).

Abbreviation: SEM, standard error of means.

The effects of different forms and levels of feather meal on carcass characteristics, including breast and thigh percentages relative to live body weight, are presented in Table 6. Supplementing the diet with various forms of feather meal had no significant effect on the percentage of abdominal fat, liver weight, or spleen weight (*p* > 0.05). However, the percentage of carcass yield, as well as breast and thigh yields, decreased in birds fed diets containing 33% and 67% boiled feather meal (33BFM, 67BFM) compared to the control group (C) (*p* < 0.05). No significant differences were observed in the percentage of carcass, breast, or thigh yield between the control diet and diets containing 33% hydrolyzed feather meal with enzymes (33HFME) or with *Streptomyces* bacteria (33HFM‐S) (*p* > 0.05). However, when the inclusion level of hydrolyzed feather meal was increased to 67%, the percentage of carcass, breast, and thigh yield significantly decreased compared with the control diet (*p* < 0.05). Additionally, hydrolyzed feather meal with enzymes (33HFME) led to better performance than hydrolyzed feather meal with *Streptomyces* bacteria (33HFM‐S), although the difference was not statistically significant (*p* > 0.05)

**TABLE 6:** The effect of different forms and levels of feather on carcass in broilers.

Dietary treatments									
		Feather meal		Enzymatic feather		Bacterial feather			
Item (%)	Control	33	67	33	67	33	67	SEM	*p* value
Carcass	70.60a	68.50bc	68.37c	70.23ab	68.74bc	70.55a	68.61bc	0.27	0.02
Fat	2.18	2.19	2.22	2.23	2.19	2.19	2.25	0.009	0.31
Breast	24.83a	23.14b	23.26b	24.76a	23.50b	24.91a	23.33b	0.21	0.01
Tights	17.47a	16.08b	16.40b	17.40a	16.43b	17.39a	16.42b	0.13	0.000
Liver	1.60	1.65	1.69	1.62	1.64	1.56	1.66	0.012	0.15
Spleen	016	0.13	0.14	0.14	0.15	0.15	0.14	0.002	0.12

*Note*: In each row, means with different superscripts are significantly different (*p* < 0.05).

Abbreviation: SEM, standard error of means.

As shown in Table 7, The digestibility of fat, ash, calcium, and phosphorus in the ileal contents was not significantly affected by dietary treatments (p > 0.05). However, there were significant differences in the digestibility of dry matter and protein depending on the type and level of feather meal used in the diets.

**TABLE 7:** The effect of different forms and levels of feather on nutrient digestibility in broilers.

Dietary treatments									
		Feather meal		Enzymatic feather		Bacterial feather			
Item (%)	Control	33	67	33	67	33	67	SEM	*p* value
Dry matter	91.19a	87.25c	87.40c	91.08a	88.53bc	90.02ab	87.35c	0.42	0.001
Protein	70.19a	64.13b	65.03b	70.36a	65.30b	70.19a	64.97b	0.77	0.02
Fat	73.15	72.13	73.52	78.80	76.85	74.60	75.21	1.08	0.73
Ash	51.54	51.57	54.01	55.07	54.91	50.60	53.75	1.18	0.94
Calcium	47.09	47.12	48.30	49.16	49.96	50.78	45.41	1.19	0.95
Phosphorus	43.00	43.02	42.68	43.23	45.96	44.87	43.51	1.09	0.99

*Note*: In each row, means with different superscripts are significantly different (*p* < 0.05).

Abbreviation: SEM, standard error of means.

For the BFM treatments, diets containing 33% (33BFM) and 67% (67BFM) boiled feather meal significantly decreased the ileal digestibility of dry matter and protein compared to the control (C) diet (*p* < 0.05). In contrast, diets containing hydrolyzed feather meal with enzymes (33HFME) or hydrolyzed feather meal with *Streptomyces* bacteria (33HFM‐S) at the 33% inclusion level showed no significant difference in digestibility compared to the control (*p* > 0.05). At the 67% inclusion level, both hydrolyzed feather meal with enzymes (67HFME) and hydrolyzed feather meal with *Streptomyces* bacteria (67HFM‐S) resulted in significantly lower digestibility of dry matter and protein compared to the control (*p* < 0.05). Finally, while the hydrolyzed feather meal with enzymes (33HFME) showed better digestibility than the hydrolyzed feather meal with *Streptomyces* bacteria (33HFM‐S), this difference was not statistically significant (*p* > 0.05).

## 4 Discussion

The crude protein content and pepsin digestibility of hydrolyzed feather meal treated with enzyme and *Streptomyces* sp. MSM5 were significantly higher than those of untreated feather meal (*p* < 0.05). This indicates that the treatments effectively enhanced both the digestibility of the protein and its overall availability. The improvement in protein availability can be attributed to the breakdown or modification of disulfide bonds that crosslink the keratin proteins in feather meal. Keratin, the primary protein in feathers, is held together by these covalent bonds, which are formed between sulphur‐containing amino acids such as cysteine. These disulfide bonds contribute to the rigidity and resistance of keratin to enzymatic degradation. By disrupting these disulfide bonds, the treatments likely increased the accessibility of the keratin protein to digestive enzymes like pepsin.

This enhancement in digestibility suggests that the treated feather meal becomes more accessible to the digestive system, making the protein more available for absorption and improving its nutritional value for poultry. Furthermore, the increased protein digestibility and availability resulting from the treatment are important for improving the nutritional quality of feather meal. As a high‐protein, cost‐effective ingredient, feather meal has the potential to be a valuable component in poultry diets. However, its benefits depend on its digestibility, which the treatments appear to significantly enhance. These findings suggest that the treatments used in this experiment could be a promising method to improve the use of feather meal in poultry feed, offering both a more digestible protein source and better nutritional value for poultry growth.

Feather protein has been identified as a valuable source of metabolizable protein in animal feed. Studies have shown that bacterial‐treated feathers have nutritional profiles comparable to soybean meal (Williams et al. 1991). While the protein in feathers is beneficial, supplementation with certain amino acids may still be required to meet the full nutritional needs of animals.

One promising approach to improving the digestibility of feather protein is the use of microbial keratin hydrolysates. Research has demonstrated that combining feather meal with keratinase—an enzyme that breaks down keratin—can significantly enhance protein and amino acid digestibility (Odetallah et al. 2003). This suggests that microbial keratinase could be a valuable tool for improving feather meal as an ingredient in animal feed.

Steiner, Kellems, and Church (1983) investigated the effects of treating feathers with different concentrations of sodium hydroxide (NaOH) or phosphoric acid (H₃PO₄). They found that these treatments improved the pepsin digestibility of feathers in vitro, making the protein more accessible for digestion. Similarly, Kim, Lorenz, and Patterson (2002) showed that prolonged incubation of feathers with NaOH increased feather solubility. When combined with enzyme treatment, feather solubility and pepsin digestibility were further improved.

In addition to chemical treatments, enzymatic methods have also been explored to enhance feather digestibility. Papadopoulos (1985) used NaOH and maxatase enzymes to treat broiler feathers, which resulted in the cleavage of cystine disulfide bonds. This process increased feather solubility and made the protein more susceptible to digestive enzymes, improving its overall nutritional value.

More recent studies have focused on the role of specific keratinases in improving feather protein quality. For example, Eaksuree et al. (2016) reported that keratinases from *Bacillus licheniformis* KUBK0006 (K6) and *Bacillus pumilus* KUB‐K0082 (K82) enhanced the protein quality and pepsin digestibility of feather meal. These enzymes, combined with hydrothermal treatments, demonstrated promising results in improving the digestibility and nutritional value of feather protein (Adler et al. 2018; Pfeuti et al. 2019).

*Streptomyces* sp. MSM5 has been shown to produce keratinase with impressive results: 23.43 U/mL enzyme activity, a 67.64% degree of degradation, and a 2.5‐cm zone of clearance on feather agar meal. These findings suggest that *Streptomyces* sp. MSM5 is highly suitable for large‐scale keratinase production.

In a similar vein, Haq, Akram, and Jabbar (2020) demonstrated that *Bacillus* sp. NKSP‐7, isolated via submerged fermentation, produces efficient extracellular keratinase capable of degrading persistent poultry feather waste. Likewise, Nnolim, Okoh, and Nwodo (2020) isolated *Bacillus* sp. Nnolim‐K1 from dumpsite soil, which exhibited high keratinolytic activity. These findings align with the work of Abdelmoteleb et al. (2023), who investigated keratinase activity in *Streptomyces netropsis* A‐ICA and *Bacillus subtilis* ALICA, both isolated from desert plant rhizospheres. Collectively, these studies underscore the potential of these strains in converting chicken feathers into soluble proteins and other useful products.

Further research has explored the practical applications of keratinase in animal feed. Adejumo and Adetunji (2018) examined the use of enzyme‐degraded feather meal from *Bacillus subtilis* in broiler diets. Their study found that the enzyme‐degraded feather meal was not only safe, but also had potential for improving broiler performance with slight feeding adjustments. Notably, the use of immobilized enzyme‐degraded feather meal showed enhanced performance compared to the control.

In another study, Abd El‐Aziz, Khalil, and Ibrahim (2023) investigated keratinase production among various actinomycetes strains, identifying *Streptomyces werraensis* KN23 as the top producer with an enzyme activity of 51.60 U/mL. Their research also highlighted significant advancements in keratinase production using a mutant strain, SA‐27, which demonstrated promise for diverse industrial applications, particularly in food‐related industries.

Larasati et al. (2017) isolated a thermophilic *Bacillus* strain, *Bacillus* sp. SLII‐1, which produced keratinase with high activity and protein content. This strain was able to increase the water‐soluble protein content in feather waste by up to 22.06%. Similarly, Jain et al. (2016) and Kumar, Kazi, and Azim (2023) demonstrated that enzymatically treated chicken feather waste could replace a substantial portion of soybean meal protein in broiler diets, leading to improved broiler performance compared to conventional soybean meal sources.

The amino acid composition of feather meals was analyzed in this study. The levels of alanine, cysteine, glycine, serine, and valine in hydrolyzed feather meal treated with enzyme and *Streptomyces* sp. MSM5 were significantly higher than those found in soybean and boiled feather meal. Overall, hydrolyzed feather meal with enzymes and *Streptomyces* sp. MSM5 showed better amino acid profiles than boiled feather meal. These findings suggest that enzyme treatment and *Streptomyces* sp. MSM5 can enhance the amino acid content of feather meal.

This study analyzed the amino acid composition of feather meal and found that hydrolyzed feather meal, treated with enzymes and *Streptomyces* sp. MSM5, had significantly higher levels of alanine, cysteine, glycine, serine, and valine compared to soybean and boiled feather meal. These results suggest that enzyme treatment and *Streptomyces* sp. MSM5 can improve the amino acid profile of feather meal, making it a more viable protein source. Feather meal has been identified as a promising alternative to traditional protein sources, such as soybean meal, in animal diets. Hasni et al. (2014) demonstrated that hydrolyzed feather meal can effectively replace up to 30% of soybean meal in broiler diets without negatively impacting performance. Specifically, their study showed that substituting fish meal with feather meal at various inclusion levels did not adversely affect broiler performance. This can be attributed to the presence of essential amino acids, such as cysteine, threonine, and arginine, which help improve the amino acid profile of the diet and support overall nutritional balance.

Feather meal is an emerging alternative protein source, particularly due to its high cysteine, threonine, and arginine content, which are essential for protein synthesis and immune function. However, it has limitations as a sole protein source, as it is deficient in key amino acids such as lysine, methionine, histidine, and tryptophan, making it unsuitable for replacing conventional protein sources such as fishmeal or soybean meal in poultry diets without supplementation. To address these deficiencies, hydrolysis and microbial treatments, such as the use of *Streptomyces* sp. MSM5, can significantly improve feather meal's digestibility and amino acid profile by enhancing the bioavailability of nutrients, making it a more viable protein source. Feather meal can be used as a partial replacement for fishmeal in broiler diets, typically replacing 8%–30% of the fishmeal without negatively affecting growth performance, provided it is supplemented with the necessary amino acids. This makes feather meal a cost‐effective alternative, particularly in regions where fishmeal is expensive. However, due to its amino acid imbalances, it cannot fully replace fishmeal or soybean meal and should be included in well‐balanced formulations designed to meet the nutritional needs of poultry. Consequently, feather meal can replace fish meal without negatively impacting live performance, carcass yield, or mortality, while also reducing feed costs.

Tsang et al. (1963) found no significant difference in weight gain when 8% feather meal was included in the diet, and Wang et al. (1990) reported no differences in the final live body weight when feather meal was substituted for up to 60% of fish meal. These studies suggest that feather meal can be effectively used in poultry diets when included at appropriate levels.

In our research, the effects of different forms and levels of feather on performance were evaluated. The supplementation of the diet with various forms of feather meal did not show any significant effect on body weight, feed intake, or feed conversion ratio (FCR) during the initial 1–10 days and 11–21 days of the experiment (*p* > 0.05).

However, during the finisher period (22–42 days), and over the entire trial (1–42 days), performance was affected by certain treatments. Specifically, a decrease in BWG and a corresponding increase in FCR were observed in groups receiving diets with 33% and 67% boiled feather meal, compared to the control group (*p* < 0.05). These findings indicate that higher inclusion levels of boiled feather meal negatively impacted growth and feed efficiency during the later stages of the experiment.

For the diets containing hydrolyzed feather meal with an enzyme and *Streptomyces* sp. MSM5, results varied with the inclusion level. At a 33% inclusion level, the hydrolyzed feather meal did not differ significantly from the control in terms of BWG and FCR during the 22‐ to 42‐day and 1‐ to 42‐day periods. However, at a 67% inclusion level, there was a noticeable decrease in BWG and an increase in FCR, similar to the boiled feather meal treatments.

Furthermore, the hydrolyzed feather meal with enzyme supplementation yielded better results than the hydrolyzed feather powder with *Streptomyces* sp. MSM5, as evidenced by the improved performance at the 33% inclusion level (*p* < 0.05). This suggests that the enzyme‐enhanced hydrolyzed feather meal was more beneficial for growth and feed efficiency compared to the alternative microbial supplementation.

These findings align with earlier studies. Ochetim (1993) reported that including 4.5% feather meal in broiler diets decreased growth rate and BWG. Similarly, Bielorai et al. (1983) observed a reduction in BWG in broilers fed feather meal diets. These declines in performance are likely attributed to amino acid imbalances and the relatively low digestibility of feather meal. Feather meal is high in leucine, but deficient in isoleucine and contains more arginine than lysine. This amino acid imbalance may interfere with protein synthesis and negatively affect bird performance, as noted by previous studies (Bielorai et al. 1983; Ochetim 1993).

Decreased protein content and deficiencies in essential amino acids can have detrimental consequences on growth and feed intake. However, feather protein is considered an excellent source of metabolizable protein (Wang and Parsons 1997). While the nutritional value of keratin is limited, both its digestibility and amino acid composition can be improved through enzymatic modification with keratinase (Larasati et al. 2017). It is reported that *Bacillus* sp. SLII‐1 produces keratinase with significant enzyme activity, achieving a rate of 2.08 (mg/s)/mL and a protein content of 6.6 mg/mL. This enzyme can enhance the water‐soluble protein content of feather waste, increasing it by up to 22.06%. When feather meal hydrolyzate protein was included in broiler diets, replacing about 5% of soybean meal protein, it resulted in improved performance compared to conventional soybean meal sources (Larasati et al. 2017).

However, other studies have reported that including hydrolyzed feather meal with enzyme treatment and *Streptomyces* sp. MSM5 at a level of 33% in the diet did not significantly affect BWG or FCR compared to the control group. This suggests that the effects of these treatments on broiler performance might be linked to their ability to improve the amino acid composition of feather meal.

Enzyme treatment and the addition of *Streptomyces* sp. MSM5 are known to increase the amino acid content in feather meal. *Streptomyces* sp. MSM5, in particular, produces keratinase with high activity, measuring 23.43 (U/mL) enzyme activity and achieving a 67.64% degree of degradation on feather agar meal. This makes it possible to replace up to 33% of soybean meal protein without negatively impacting broiler performance.

The benefits of keratinase in these treatments are largely due to its ability to break down keratin by disrupting hydrogen and disulfide bonds, as described by Gaman and Sherrington (1992). This hydrolysis process improves protein absorbability and supports growth. By breaking down keratin into amino acids and peptides, keratinase enhances the digestibility of chicken feather waste and indirectly contributes to better growth performance in broilers (Mousavi et al., 2013).

Our results indicated that feeding diets containing 33% and 67% boiled feather meal led to a decrease in the percentage of carcass, breast, and thigh weights. This is in contrast to studies by Isika, Agiang, and Eneji (2006) and Ayanwale et al. (2023), who found that feeding broilers diets with up to 3% and 5% feather meal, respectively, did not significantly affect carcass weight or overall performance. Similarly, Adejumo and Adetunji (2018) observed no significant changes in the relative weights of liver, viscera, wings, or gizzard when broilers were fed biodegraded feather meal treated with enzymes.

Ochetim (1993) also reported that including feather meal in broiler diets did not impact dressing percentage. He attributed any observed differences in carcass yields to variations in final body weight at slaughter. Taken together, these findings suggest that feather meal can be incorporated into broiler diets without major negative effects on carcass characteristics.

In our study, the decrease in carcass, breast, and thigh percentages may have been influenced by the impact of feather meal on final body weight. This is consistent with our results, which showed that feather meal reduced body weight, likely due to a deficiency in essential amino acids. However, when hydrolyzed feather meal was included in the diet at 33%, along with an enzyme and *Streptomyces* sp. MSM5 treatment, no significant differences were observed compared to the control group. This suggests that the enzyme and *Streptomyces* sp. MSM5 treatments improved the amino acid composition of the feather meal, potentially mitigating its negative effects on body weight and carcass characteristics.

In the current study, the digestibility of DM and crude protein (CP) was negatively affected when broilers were fed diets containing 33% and 67% boiled feather meal or 67% hydrolyzed feather meal treated with enzymes and bacteria. However, diets containing 33% hydrolyzed feather meal with enzymes and *Streptomyces* sp. MSM5 showed similar digestibility values to the control diet for both DM and CP.

The improvement in CP digestibility observed with the hydrolyzed feather meal treatment suggests that enzymatic breakdown, aided by *Streptomyces* sp. MSM5, likely resulted in the breakdown of feather proteins into constituent amino acids. Despite this breakdown, the amino acids may not have been utilized effectively in tissue biosynthesis, possibly due to poor protein quality in the hydrolyzed feather meal. This supports the idea that while hydrolyzed feather meal with *Streptomyces* sp. MSM5 improved CP digestibility, it was not sufficient for optimal protein utilization in broiler growth.

Additionally, the increase in DM digestibility observed in birds fed hydrolyzed feather meal with enzymes and *Streptomyces* sp. MSM5 could be attributed, in part, to the enhanced digestibility of crude protein. The 33% inclusion level of hydrolyzed feather meal with *Streptomyces* sp. MSM5 appeared to be tolerable for broilers, as it did not significantly affect DM and CP digestibility. However, increasing the inclusion level to 67% led to a decrease in both DM and CP digestibility, suggesting that higher levels of hydrolyzed feather meal with *Streptomyces* sp. MSM5 might exceed the birds' ability to efficiently process the protein and dry matter in the diet.

These findings align with those of Eaksuree et al. (2016), who reported that broilers fed a diet containing 10% K82 keratinase‐treated feather meal showed improved apparent nitrogen retention compared to those fed untreated feather meal. Similarly, Aly and Tork (2018) observed higher nutrient digestibility in birds fed hydrolyzed feather meal diets compared to controls. Yeh, Hsieh, and Chen (2023) also found that fermentation with *Bacillus subtilis* var. *natto* N21 and *Saccharomyces cerevisiae* Y10 improved the amino acid digestibility of enzymatically treated feather meal.

While the digestibility of DM and CP was significantly impacted by the treatments, no significant effects were observed on the digestibility of ileal fat, ash, calcium, or phosphorus. The lack of effect on fat digestibility can be explained by the fact that hydrolyzed feather meal primarily influences protein metabolism and does not directly alter lipid digestion, which relies on enzymatic activity in the pancreas and small intestine. Similarly, the digestibility of ash, calcium, and phosphorus may not have been influenced by the dietary protein source, as these minerals are primarily absorbed in the small intestine and their digestibility is less affected by protein sources. The treatments applied in this study, including enzymatic and microbial treatments, may not have significantly altered the bioavailability of these minerals in the feather meal, leading to no observable impact on their digestibility.

The absence of significant differences in performance traits such as feed intake and body weight during the starter phase further suggests that broilers adapted well to the diets with feather meal inclusion, especially at the 33% level. This indicates that the processing methods (hydrolysis and bacterial enzyme treatment) likely mitigated potential anti‐nutritional effects of feather meal, ensuring comparable performance outcomes. The uniform basal diet composition likely ensured sufficient nutrient availability across all groups, and the partial replacement of soybean meal with feather meal, particularly at lower inclusion levels, may not have been sufficient to affect the digestibility of fat, ash, calcium, and phosphorus.

In conclusion, while the treatments showed potential improvements in CP and DM digestibility, their effects on other nutrients like fat and minerals were minimal, suggesting that these nutrients were not as influenced by the treatments. Future research could further explore how protein hydrolysis and mineral bioavailability interact to better understand the overall impact of such treatments on nutrient digestibility in broilers.

## 5 Conclusions

It was concluded from the present study that *Streptomyces* sp. is capable of producing keratinase with an enzyme activity of 23.43 U/mL and a degradation degree of 67.64% on feather agar meal. This revealed that *Streptomyces* sp. MSM5 is suitable for large‐scale keratinase production in industrial applications. Furthermore, hydrolyzed feather meal treated with the enzyme and bacteria can successfully replace 33% of soybean meal in broiler diets without impairing performance. Despite these findings, we recommend further research to isolate new keratinase‐producing actinomycete strains and apply sequential mutagenesis to enhance keratinase productivity. These efforts could play a pivotal role in reducing the environmental impact of poultry production and decreasing the industry's reliance on soybean meal. Additionally, we emphasize the need for increasing the number of replicates in future studies to improve data reliability and acceptance. Increasing the replication number would provide more robust and statistically significant results, helping to account for biological variability and strengthening the overall validity of the conclusions.

## Author Contributions

Mandana Salehizadeh contributed to the collection of samples, the design of the experiment, the analysis, and interpretation of the data, as well as writing the manuscript. Maryam Tajabadi Ebrahimi contributed to the design of the experiment, the analysis, and interpretation of the data, revision, as well as final editing. Seyed Naser Mousavi contributed to the collection of samples, the design of the experiment, the analysis, and interpretation of the data, the writing of the manuscript, revision, as well as final editing. Abbas Akhavan Sepahid contributed to the design of the experiment, interpretation of the data, the manuscript revision and final editing. Reza Orooji contributed to the analysis, and interpretation of the data. All authors read and approved the final manuscript.

## Ethics Statement

The study protocol was approved by Animal Science Research Institute of IRAN (ASRI), and the experiments were performed in accordance with the internationally accepted standard ethical guidelines for animal use and care of Iran Veterinary Organization.

## Conflicts of Interest

The authors declare no conflicts of interest.

### Peer Review

The peer review history for this article is available at https://publons.com/publon/10.1002/vms3.70199.

## Data Availability

The data supporting the finding of this study are available within the manuscript. further data can be obtained from the corresponding author upon reasonable request.

## Endnotes

1 Valkerase is a unique keratinase (an American commercial enzyme). https://briworldwide.com/products/valkerase/.
